# Developmental regulation of the neuroinflammatory responses to LPS and/or hypoxia-ischemia between preterm and term neonates: An experimental study

**DOI:** 10.1186/1742-2094-8-55

**Published:** 2011-05-20

**Authors:** Marie-Elsa Brochu, Sylvie Girard, Karine Lavoie, Guillaume Sébire

**Affiliations:** 1 Child Neurology Laboratory, Université de Sherbrooke, Canada Faculté de Médecine et des Sciences de la Santé, Université de Sherbrooke, 3001 12eme avenue Nord, J1H5N4 Sherbrooke, Canada

## Abstract

**Background:**

Preterm and term newborns are at high risk of brain damage as well as subsequent cerebral palsy and learning disabilities. Indeed, hypoxia-ischemia (HI), pathogen exposures, and associated intracerebral increase of pro-inflammatory cytokines have all been linked to perinatal brain damage. However, the developmental effects of potential variations of pro- and anti-inflammatory cytokine ratios remain unknown.

**Methods:**

Using rat models of perinatal brain damage induced by exposures to lipopolysaccharide (LPS) and/or HI at distinct levels of maturity, we compared cytokine expression at stages of cerebral development equivalent to either preterm (postnatal day 1, P1) or term (P12) newborns.

**Results:**

At P1, expression of anti-inflammatory cytokine within the brain was either not modulated (IL-6, IL-10) or down-regulated (IL-1ra, TGF-β1) by HI, LPS or LPS+HI. In contrast, there was at P12 an up-regulation of all anti-inflammatory cytokines studied in HI or LPS+HI condition, but not after LPS exposure. Interestingly, IL-1β was the main pro-inflammatory cytokine up-regulated moderately at P1, and strongly at P12, with a weak co-expression of TNF-α observed mainly at P12. These age-dependant inflammatory reactions were also accompanied, under HI and LPS+HI conditions, at P12 only, by combined: (i) expression of chemokines CINC-1 and MCP-1, (ii) blood-brain barrier (BBB) leakage, and (iii) intracerebral recruitment of systemic immune cells such as neutrophils. In contrast, sole LPS induced IL-1β responses mainly within white matter at P1 and mainly within gray matter at P12, that were only associated with early MCP-1 (but no CINC-1) induction at both ages, without any recruitment of neutrophils and CD68+ cells.

**Conclusion:**

HI and LPS+HI induce pro-inflammatory oriented immune responses in both preterm and term like brains, with a maximal inflammatory response triggered by the combination of LPS+HI. The profile of these neuroinflammatory responses presented striking variations according to age: no or down-regulated anti-inflammatory responses associated with mainly IL-1β release in preterm-like brains (P1), in sharp contrast to term-like brains (P12) presenting stronger anti-and pro-inflammatory responses, including both IL-1β and TNF-α releases, and BBB leakage. These developmental-dependant variations of neuroinflammatory response could contribute to the differential pattern of brain lesions observed across gestational ages in humans. This also highlights the necessity to take into consideration the maturation stage, of both brain and immune systems, in order to develop new anti-inflammatory neuroprotective strategies.

## Background

Human newborns, especially preterm, are at high risk of brain damage [1-4]. Decreased oxygen and other blood nutrient supply to the brain, remote pathogen exposure, or both combined, and the associated neuroinflammatory responses are the most important perinatal risk factors associated with brain injury and subsequent cerebral palsy and/or learning and behavioral impairments [1-3]. The incidence of these forms of neonatal brain damage is inversely proportional to gestational age and thus higher in preterm than in term newborn [4]. Type and distribution of brain lesions differ markedly between preterm and term newborns [4,5]. This is attributed to different levels of brain maturity and vulnerability to aggression due to regional and age-specific metabolic needs [1,2,4,5]. Post-natal bacterial infections - that are more frequent in pre-term than term newborns -, and often combined with hypoxia-ischemia (HI), are also associated with an increased risk to develop brain lesions [6]. Our hypothesis is that developmental differences in neuroinflammatory responses contribute to the age-specific patterns of brain injury.

Pro-inflammatory cytokine expression within the brain, especially IL-1β and TNF-α, has been implicated in perinatal brain damage induced by pathogen components and/or HI both in experimental model [7-12] and the human newborn brain [13-23]. On the other hand, relatively little is known about anti-inflammatory and neurotrophic cytokine responses in such perinatal brain damage. Anti-inflammatory cytokines, such as IL-1 receptor antagonist (IL-1ra), IL-6, IL-10, and TGF-β1, are already known either to be constitutively expressed to support brain development, or to be induced in pathological conditions to counterbalance the pro-inflammatory response and to promote neuronal survival [24-34]. Although IL-6 is often classified as a pro-inflammatory mediator in peripheral pathologies, it has not been directly implicated in perinatal brain damage and its blockage was even showed to be deleterious in some pathological circumstances [35,36]. During brain development, IL-6 has established neurotrophic properties [35].

To uncover potential differences between term and preterm neuroinflammatory responses to neonatal insults, we used rat models of brain damage induced at different stages of brain development. Postnatal day 1 (P1), corresponds, in term of brain development, to the very preterm human brain (26-32 weeks of gestation), whereas P12, corresponds to the term human neonate brain [37]. We compared the intracerebral profiles of pro- and anti-inflammatory cytokines responses, resulting chemokines responses, and related immune cell recruitments at both developmental stages.

## Methods

### Animals

Lewis dams were purchased from Charles River Laboratories (Saint-Constant, Quebec, Canada) at embryonic day 16 (E16) and gave birth naturally. At P1 or P12, rat pups were injected intraperitoneally (ip) either with lipopolysaccharide (LPS; 200 μg/kg, *Escherichia coli*, 0127:B8; Sigma, ON, Canada) diluted in 50 μl of saline, or saline only. HI was induced 4 h after the LPS injection by permanent ligation of the right common carotid artery followed by exposure to 8% O_2_/N_2 _for 210 min at P1 or 90 min at P12 in a chamber at 36°C as described previously [38]. The duration of hypoxia was decreased at P12 because of higher mortality rates of pups during hypoxia than at P1. The P1 and P12 pups were randomized into five groups: (1) control, (2) sham, (3) HI, (4) LPS, (5) LPS+HI. Pups were sacrificed by decapitation at 4, 24, 48 h and 8 days post-HI and brain were either frozen and kept at -80°C for protein extraction or fixed in paraformaldehyde and embedded in paraffin for immunohistochemistry (IHC) (see below for details). Since there was no difference between control and sham animals, we presented only control group in our figures. This protocol was duly approved by the appropriate institutional Animal Research Ethics Board and conducted in accordance with all applicable laws and regulations.

### Brain cytokines quantification

Both cerebral hemispheres were studied in combination at P1 due to the presence of bilateral and symmetrical brain damage, and of similar level of cytokine expression (by IHC) between ipsi- and contralateral hemispheres in animal exposed to LPS+/-HI at P1 as already shown in HI condition [38], and in the present work in other conditions (see Result Section). At P12, cerebral hemispheres were studied separately since brain damage was mostly unilateral. Proteins were extracted and concentrations determined as previously described [39]. Cytokines (IL-1β, TNF-α, IL-10, IL-1ra, IL-6, TGF-β1) and chemokines (MCP-1 and CINC-1) were quantified using ELISA kits according to manufacturer's instruction (R&D System, MN, US; except for MCP-1: BD Biosciences, NJ, US). ELISA for IL-1ra detection was performed as described previously [7]. All samples were analyzed in duplicate. Western blotting was used to detect specific variation in the mature form of IL-1β as previously described [39].

### Immunohistochemistry

Total brains were embedded in paraffin and 5 μm-thick sections were mounted on silanized slides. Brain sections were prepared as previously described [39]. Sections were incubated overnight at 4°C with the following primary antibodies directed against the following proteins: IL-1β (1:50; #AAR15G, Serotec, NC, US), TNF-α (1:50; #AB1837P Chemicon, ON, Canada), IL-10 (1:10; #MAB519, R&D System, MN, US), IL-1ra (1:25; #sc-25444, Santa Cruz Biotechnology, CA, US), IL-6 (1:100; #MAB5061, R&D System, MN, US), TGF-β1 (1:100; #sc-146, Santa Cruz Biotechnology, CA, US), MCP-1 (1:50; #AB1834P, Chemicon, ON, Canada), CINC-1 (1:50; #CLLS-B2513, LifeSpan Biosciences, WA, US), CD68 (1:250; #MAB1435, Chemicon, ON, Canada), and with adsorbed antiserum directed against rat neutrophils (1:100; #CLAD51140, Cedarlane, ON, Canada). For albumin detection, sections were incubated 2 h at room temperature with IgG albumin fraction (1:100; #55727, MP Biomedicals, OH, US) after blocking overnight with 10% milk. The appropriate HRP-conjugated secondary antibodies (anti-mouse, Cell Signaling Technology, MA, US; anti-rabbit, Serotec, NC, US; anti-swine, Cedarlane, ON, Canada) were used for each primary antibody and incubated for 1 h at room temperature. Labeling was revealed using diaminobenzidene (DAB) (Roche, Qc, Canada). Slides were counterstained with hematoxylin. Cytokine and chemokine staining intensities were measured in brain cortex and striatum, both combined and referred to as gray matter, and in adjacent subcortical/periventricular white matter (corpus callosum and external capsule) using Image J program (NIH) as described previously [23]. Quantitative comparisons were performed between staining intensities from LPS +/- HI conditions compared to control. In our IHC graphs, fold increases were determined against the mean of the control group for each animal in each experimental condition; data presented are thus the mean+/-SEM fold variation from 3 to 4 animals in each given experimental conditions. Neutrophils and CD68+ cells were counted in coronal sections at bregma level -0.36 mm in the brain hemisphere ipsilateral to ischemia, as previously described [40].

### Statistical analyses

Data are presented as mean ± standard error of the mean (SEM). Comparisons between groups were performed using one-way analysis of variance (ANOVA) with the Student-Newman-Keuls post test and the unpaired t-test with Welch correction. The significance level was set at p < 0.05.

## Results

### A. Comparison of brain anti-inflammatory responses at P1 and P12 after LPS and/or HI exposure

**Figure 1:**
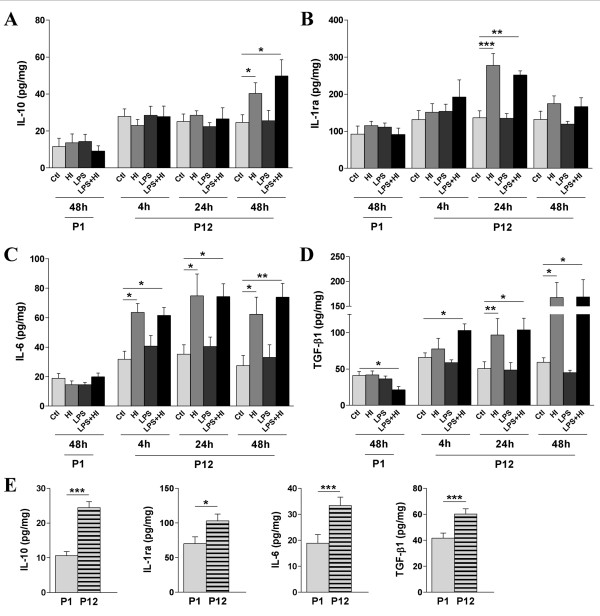
**Comparison between P1 and P12 ELISA titrations of brain anti-inflammatory cytokines**. Developmentally regulated induction of anti-inflammatory cytokines was shown after exposure to HI +/- LPS. An increased expression was detected after LPS and/or HI exposures at P12, but not at P1, for IL-10 (**A**), IL-1ra (**B**), IL-6 (**C**) and TGF-β1 (**D**). At P1, since the induction pattern was the same at 4, 24 or 48 h post-HI, only 48 h was shown. Control levels (**E**) of intracerebral cytokine titers were compared between P1 and P12. Protein detection was performed on 3 to 6 brains assayed in duplicate at each time point under each experimental condition. *p < 0.05, **p < 0.01, ***p < 0.001, one-way ANOVA with Newman-Keuls post test (**A-D**) and t test with Welch correction (**E**).

We first assessed the impact of exposures to LPS and/or HI on the expression of anti-inflammatory cytokines in P1 and P12 pup brains. We quantified IL-10, IL-1ra, IL-6 and TGF-β1 expressions in cerebral hemispheres by ELISA. In P1 brain, LPS and/or HI did not induce the expression of any of the anti-inflammatory cytokines studied (IL-10, IL-1ra, IL-6 and TGF-β1) at 4 h, 24 h, or 48 h post-HI (the latter shown in Figure 1A-D). Brain from P1 rat pups exposed to combined LPS+HI presented a decreased expression of TGF-β1 (Figure 1D) compared to control level. In contrast, brain from P12 pups exposed to HI and LPS+HI, had up-regulated levels of all anti-inflammatory cytokines in the hemisphere ipsilateral to ischemia, as compared to control condition (Figure 1A-D); HI and LPS+HI contralateral hemisphere presenting similar levels of expression of anti-inflammatory cytokine as control (data not shown). The induction of IL-6 and TGF-β1 at P12 was detected as early as 4 h post-HI and maintained until 48 h (Figure 1C, D), expressions peaking at 48 h. On the other hand, IL-1ra and IL-10 expressions at P12 were delayed, with a transient IL-1ra increase at 24 h (Figure 1B), and up-regulation of IL-10 at 48 h post-HI (Figure 1A). Administration of LPS prior to HI did not lead, at P12, to any further increase of any anti-inflammatory cytokines (IL-10, IL-1ra, IL-6, TGF-β1) compared to HI alone.

The weaker induction of anti-inflammatory cytokines at P1 compared to P12, *i.e*. at a developmental stage corresponding to preterm versus term human brains, likely reflects the immaturity of the intracerebral immune response of the premature brain. This is further supported by the basal intracerebral expression of anti-inflammatory cytokines, which was also developmentally regulated. Indeed, constitutive levels of expression were higher at P12 than at P1 for all anti-inflammatory cytokines studied (Figure 1E).

**Figure 2:**
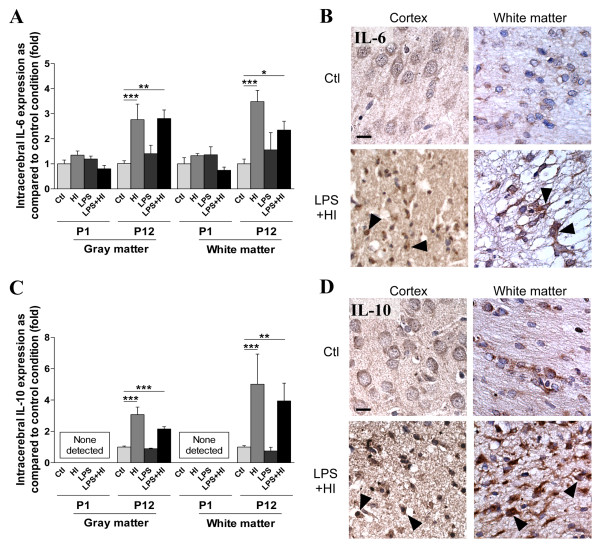
**Comparison between P1 and P12 immunostaining intensities of IL-6 and IL-10 in brain**. IL-6 and IL-10 expressions were increased only at P12 in brains of rat pups exposed to HI+/- LPS. Data are presented (**A, C**) as fold increase of IL-6 and IL-10 expressions compared to control (set at 1). (**B**) Increased IL-6 staining (arrowheads) in P12 spongiotic frontal cortex (pyknotic neurons with abnormal reduced contrast between nucleus and cytoplasm), and spongiotic underlying external capsule following LPS+HI exposure compared to control. (**D**) Increased IL-10 staining (arrowheads) in P12 lesioned frontal cortex and underlying external capsule exposed to LPS+HI compared to control. IHC was performed at 48 h post-HI, in 3 to 4 brains under each experimental condition. *p < 0.05, **p < 0.01, ***p < 0.001, one-way ANOVA with Newman-Keuls post test. Scale bars = 15 μm (**B**, **D**)

**Figure 3:**
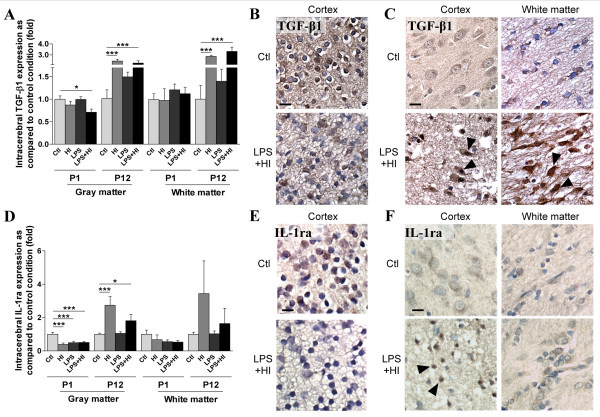
**Comparison between P1 and P12 immunostaining intensities of TGF-β1 and IL-1ra in brain**. TGF-β1 and IL-1ra expressions were decreased at P1 and conversely increased at P12 in brains of rat pups exposed to HI and/or LPS. Data are presented (**A, D**) as fold increase of TGF-β1 and IL-1ra expressions compared to control (set at 1). Decreased TGF-β1 (**B**) and IL-1ra (**E**) staining in P1 frontal cortex exposed to LPS plus HI versus control. Increased TGF-β1 (**C**) and IL-1ra (**F**) staining (arrowheads) in P12 lesioned frontal cortex (pyknotic neurons with abnormal reduced contrast between nucleus and cytoplasm), and underlying external capsule exposed to LPS+/-HI versus control. IHC was performed at 48 h post-HI, in 3 to 4 brains under each experimental condition. *p < 0.05, ***p < 0.001, one-way ANOVA with Newman-Keuls post test. Scale bars = 15 μm (**B, C, E, F**).

We then studied, by *in situ *IHC, the localization of cytokine expression in different brain region, with a special focus on those affected in human perinatal brain damage, *i.e*. superficial (cortex) and deep (striatum) gray matter, and adjacent white matter, at 48 h (Figure 2A-D). At P1, no difference was detected in both gray and white matter, in IL-6 (Figure 2A) and IL-10 (Figure 2C) staining intensity under LPS and/or HI conditions compared to control. At P12, HI and LPS+HI increased IL-10 expression in both hemispheric gray and white matters ipsilateral to ischemia (Figure 2C, D). IL-6 expression (Figure 2A, B) at P12 followed a pattern similar to IL-10 but was increased in both ipsi- and contralateral white matter under HI and LPS+HI conditions as compared to control (data not shown). TGF-β1 expression at P1 was down-regulated in the gray matter of rat exposed to LPS+HI (Figure 3A, B). In contrast, at P12, TGF-β1 up-regulation was detected in both gray and white matters ipsilateral to ischemia (Figure 3A, C) as well as in white matter contralateral to ischemia. Intracerebral IL-1ra expression was decreased in P1 pups 48 h after exposure to HI, LPS or both combined (Figure 3D, E). Conversely, an IL-1ra increase was detected in P12 rats at 48 h post-HI and LPS+HI, but only in the gray matter of the hemisphere ipsilateral to ischemia (Figure 3D, F).

### B. Comparison between P1 and P12 intracerebral pro-inflammatory responses to LPS and/or HI

**Figure 4:**
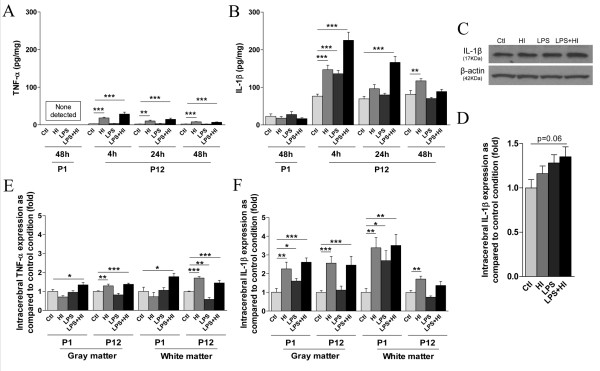
**P1 versus P12 ELISA titrations and immunostaining intensities of cerebral pro-inflammatory cytokines, IL-1β and TNF-α**. TNF-α was not detected by ELISA at P1 but an up-regulation - weaker than the one observed for IL-1β - was detected at P12 after HI and LPS+HI exposures by ELISA (**A**) and IHC in both white and gray matters (**E**). No modulation of combined pro- and active forms of IL-1β was detected by ELISA between the different experimental conditions at P1 (**B**), whereas the active form (17 kDa) of IL-1β as detected by western blot was increased in LPS+HI condition (**C, D**). Some foci of increased IL-1 immunostaining were detected by IHC in brain exposed to HI or LPS+HI, at 48 h post-HI (**F**). At P12, IHC results confirmed an increased IL-1β expression localized especially in gray, and in a lesser extent in white matter, of brains exposed to HI and LPS+HI (**F**) and a TNF-α increase was detected only in the LPS+HI brains (**E**) at P1. Protein detection was performed by ELISA on 3 to 4 brains assayed in duplicate, at each time point under each experimental condition. IHC and western blot were performed at 48 h post-HI, in 3 to 4 brains in each experimental condition. *p < 0.05, **p < 0.01, ***p < 0.001, one-way ANOVA with Newman-Keuls post test, except for LPS versus control at P1 in gray matter using t test with Welch correction.

The balance between pro and anti-inflammatory mediators and their interactions is known to determine the magnitude of the inflammatory reaction. Thus, in addition to anti-inflammatory responses, we also used ELISA to study neurodevelopmental modulation of expression of pro-inflammatory cytokines, namely IL-1β and TNF-α, following LPS and/or HI exposures. At P1, we detected no TNF-α or modulation of total IL-1β levels by ELISA in the brain (Figure 4A, B). However, using western blotting to further distinguish between pro and active forms of IL-1β, we detected a slight increase of the active form of IL-1β (17 kDa) in brains exposed to LPS+HI (Figure 4C, D). In contrast, both IL-1β and TNF-α were up-regulated (IL-1β at a much higher level than TNF-α) in P12 brains exposed to HI and LPS+HI at 4, 24 and 48 h post-HI (Figure 4 A, B).

Using IHC, we then studied the distribution of pro-inflammatory cytokines. Some rare foci of slight TNF-α expression were detected by IHC at P1, only after combined exposures to LPS+HI, in both gray and white matter at 48 h (Figure 4E). At P12, TNF-α expression was slightly increased after exposure to HI or combined LPS+HI at 4 h and 48 h post-HI in both gray and white matter (Figure 4E). IL-1β expression was also induced at P1, in both gray and white matter of animals exposed to HI and LPS+HI, and also in animals exposed to LPS alone, but mainly in white matter (Figure 4F). At P12, increased IL-1β immunoreactivity was detected at 48 h in ipsilateral gray and white matters after HI or LPS+HI (Figure 4F) as well as in contralateral white matter, albeit to a lesser extent under the same experimental conditions (data not shown).

### C. Neuropathological changes, BBB permeability and systemic immune cell recruitment

**Figure 5:**
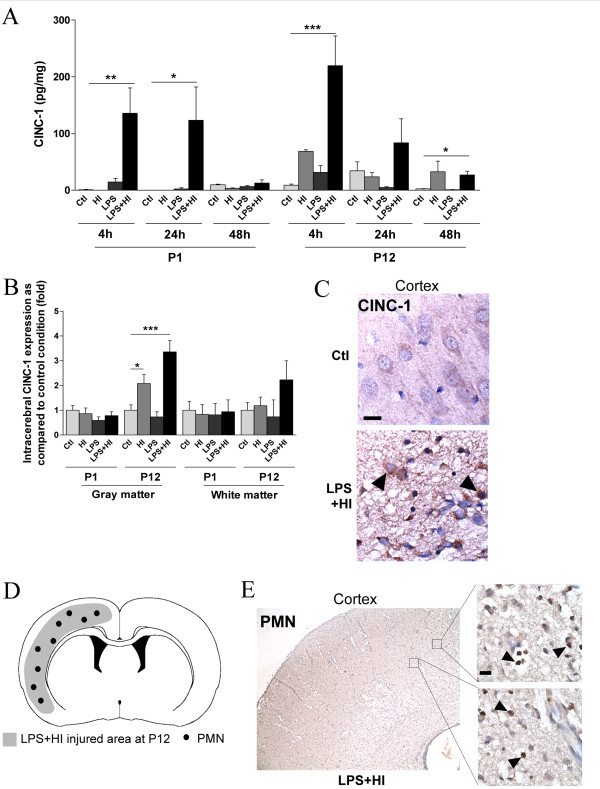
**P1 versus P12 intracerebral expressions of CINC-1 and levels of neutrophil infiltrations**. CINC-1 was detected by ELISA at 4 and 24 h after HI in P1 brains exposed to LPS+/-HI (**A**). No CINC-1 was detected at 48 h after HI in P1 brains exposed or unexposed to LPS+/-HI either by ELISA (**A**) or IHC (data not shown) at 48 h post-HI. In contrast, following HI or LPS+HI at P12, CINC-1 was up-regulated in brains at 4, 24 and 48 h post-HI as shown by ELISA (**A**) and IHC experiments (**B, C**); this increase of CINC-1 intracerebral expression (**C**, arrowheads) at P12 was present in the neocortical gray matter (**C**), but not in the underlying white matter. Neutrophils (**E**, arrowheads) were detected only at P12 - not at P1 -, at 48 h post LPS+HI, in the neocortex ipsilateral to ischemia, *i.e*. in the areas of neocortical damage as illustrated in a schema (**D**). Protein detection was performed by ELISA on 3 to 4 brains assayed in duplicate at each time point under each experimental condition. IHC was performed at 48 h post-HI, in 3 to 4 brains under each experimental condition. *p < 0.05, **p < 0.01, ***p < 0.001, one-way ANOVA with Newman-Keuls post test, except for LPS+HI versus control at P12 (48 h) by ELISA using t test with Welch correction (**A**).Scale bars = 15 μm (**C, E)**.

**Figure 6:**
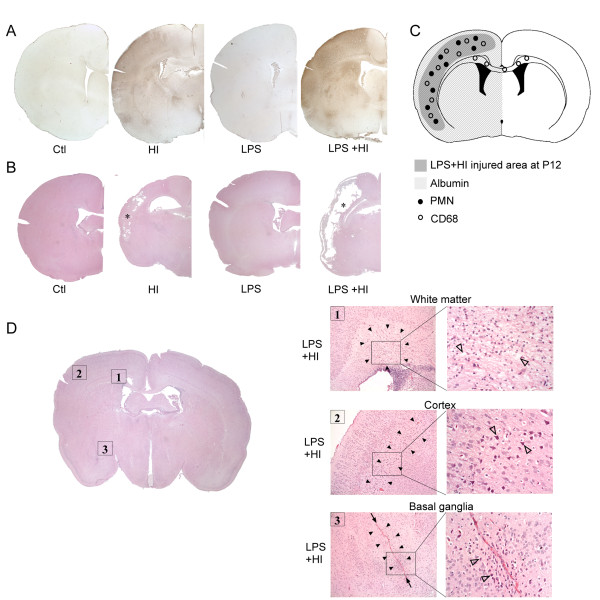
**BBB leakage and brain damage**. Distribution of increased albumin staining within right hemispheric gray and white matters exposed to HI, LPS or LPS+HI in P12 brains compared to control **(A) **correlated with injured areas **(B, C)**. Brain injuries induced by HI and LPS+HI at P12 corresponded to infarcted areas (*) located in right carotidian territory; extent severity of infarct were maximum under LPS+HI compared to HI (**B**). Schematic presentation of distribution of BBB leakage at P12, including albumin extravasations, PMN and CD68+ cells infiltrations in HI and LPS+HI brains showing a topographic association between all these components of the neuroinflammatory response and brain damage **(C)**. Brain damage at P1 was detected in both white and gray matter (cortex and basal ganglia) (**D**). In contrast to P12 lesion, P1 brains submitted to HI and LPS+HI presented laminar (**D2**, black arrowheads) or columnar (**D3**, black arrowheads) areas of neuronal necrosis (**D2, D3**, empty arrowheads), linear microhemorrhage (**D3**, arrows) and foci of white matter damage (**D1**, black arrowheads) combining spongiotic cavities **(D1**, empty arrowheads) and disorganization of cellular architecture (**D1**).

A systemic contribution of infiltrating macrophages and neutrophils to the neuroinflammatory response has been associated with HI induced brain damage, especially in adult models. We therefore studied the intracerebral recruitment of circulating immune cells and the expression of related chemokines. Using ELISA, we quantified the intracerebral expression of CINC-1 and MCP-1, which are known to mediate the recruitment of neutrophils and macrophages respectively. CINC-1 expression was induced in both P1 and P12 pups brains after exposure to LPS+HI at 4 h, and, to a lesser extent only at P12 at 48 h post-HI (Figure 5A). However, this CINC-1 induction was greater in P12 than P1 brains (2 fold higher). Those results were confirmed by IHC showing at P12 - but not at P1 - a sustained induction of CINC-1, in the gray matter, at 48 h post HI and LPS+HI (Figure 5B, C). Infiltrating neutrophils were detected at 48 h (but not at 4 h) after the aggressions, in P12 brains exposed to HI (mean number of cells counted in one section from ipsilateral hemisphere: 35 ± 28) or LPS+HI (mean number of cells counted in one section from ipsilateral hemisphere: 110 ± 45) (Figure 5D, E). In contrast, no neutrophils were detected in P1 brain exposed to LPS and/or HI. No neutrophil was detected in the hemisphere contralateral to ischemia at P12 in any of the experimental conditions used. Neutrophils were distributed in the lesioned area (mainly in gray matter of the hemisphere ipsilateral to ischemia). The highest neutrophil density - observed in LPS+HI condition - was associated with both the highest level of CINC-1 expression within gray matter, and the most extended histological brain damage (Figure 5D, 6B and Table 1).

**Table 1:** Respective neuroinflammatory and neuropathological effects observed under each experimental condition in preterm-like versus term-like brains.

Insult	P1 preterm-like forebrain		P12 term-like forebrain	
	**Pattern of neuroinflammatory response**	**Neuropathological correlate**	**Pattern of neuroinflammatory response**	**Neuropathological correlate**
	
**LPS**	IL-1β (*weak*) increase with parallel IL-1ra decrease in gray matter; lack of modulation of other pro- (TNF-α) or anti-inflammatory cytokines. MCP-1 induction (gray & white matter) likely via IL-1β/IL-1ra unbalance.	No visible change by hematoxylin staining.	*Transient *(4 h) and diffuse IL-1β (*high*) increase with dyscoordinated TNF-α decrease (48 h) predominant in white matter. No modulation of anti-inflammatory cytokines. Transient (4 h) MCP-1 increase, mainly in gray matter, likely via IL-1β.	No visible change seen by hematoxylin staining.
**HI**	IL-1β (*weak*) increase (gray & white matters) with parallel IL-1ra decrease (gray matter). No modulation of other pro- (TNF-α) or anti-inflammatory cytokines. No modulation of MCP-1 and CINC-1; no PMN or CD68+ cell infiltration.	Slight degree of laminar or columnar neuronal necrosis in neocortex and basal ganglia; foci of white matter damage. Possible direct and indirect neurotoxicity of IL-1β.	*Sustained *(4-48 h) IL-1β (*high*) and TNF-α (*weak*) responses in gray & white matter with late (24-48 h) increase of anti-inflammatory cytokines in both gray & white matter. MCP-1 (4-24 h; *high*) and CINC-1 (48 h; *moderate*) inductions (possibly via IL-1β), only in gray matter & subsequent PMN and CD68+ cell infiltration (*moderate*).	Infarcted areas in neocortex and underlying white matter. Possible neurotoxic effects of PMN and/or IL-1β.
**LPS +HI**	IL-1β (*moderate*) and TNF-α (*weak*) increase (predominant in white matter) with parallel decrease of TGF-β1, IL-1ra (gray matter) and IL-6 (diffuse). No IL-10 response. *Brief *CINC-1 and MCP-1 inductions (*high*) in gray matter, likely via IL-1β, without any PMN or CD68+ cell infiltration.	Compared to HI, more severe and abundant laminar or columnar areas of neuronal necrosis in neocortex and basal ganglia. Foci of white matter damage. Possible direct and indirect neurotoxicity of IL-1β.	*Sustained *(4-48 h) IL-1β (*very high*) and TNF-α (*moderate*) responses with late (24-48 h) increase of all anti-inflammatory cytokines in both gray & white matter. MCP-1 and CINC-1 increase (*very high and sustained *(4-48 h)), possibly IL-1-induced, within gray matter & subsequent PMN and CD68+ cell infiltrations (*high*).	Compared to HI, more extended infarcted areas in neocortex and adjacent white matter areas. Possible neurotoxic effects of PMN and/or IL-1β.

**Figure 7:**
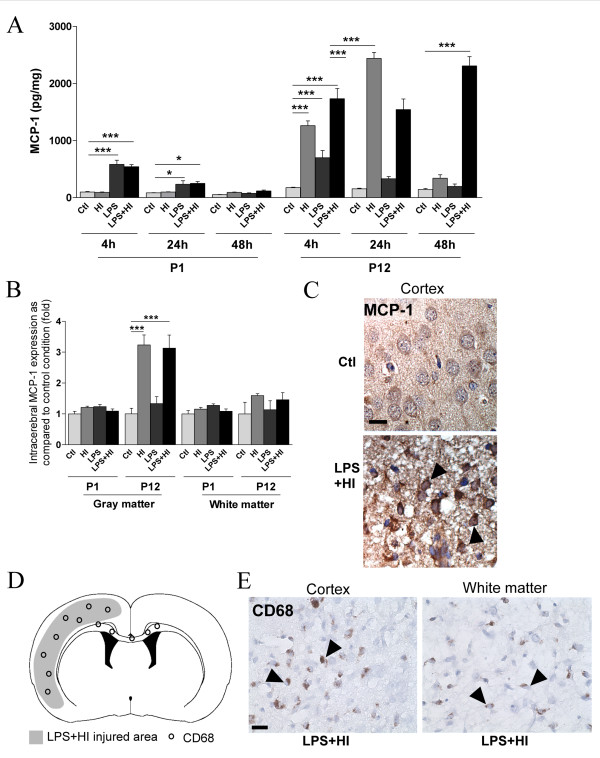
**P1 versus P12 intracerebral expressions of MCP-1 and levels of CD68+ cells**. MCP-1 was detected at 4 and 24 h post-HI in pups exposed to LPS +/-HI. No MCP-1 was detected 48 h after HI at P1 in brains exposed or unexposed to LPS+/-HI either by ELISA (**A**) or IHC (**B**) at 48 h post-HI. In contrast, following HI or LPS+HI at P12, MCP-1 was up-regulated in brains at 4, 24 and 48 h post-HI as shown by ELISA (**A**). IHC experiments (**B, C**) showed that this increased intracerebral MCP-1 expression (**C**, arrowheads) at P12 was only detected in the neocortex (**C**). CD68+ cells (**E**, arrowheads) were detected at P12 - not at P1 - at 48 h post LPS+HI in the neocortex and white matter ipsilateral to ischemia, *i.e*. in the areas of neocortical damage as illustrated in the schema (**D**). Protein detection was performed by ELISA on 3 to 4 brains assayed in duplicate at each time point under each experimental condition. IHC was performed at 48 h post-HI, in 3 to 4 brains under each experimental condition. *p < 0.05, **p < 0.01, ***p < 0.001, one-way ANOVA with Newman-Keuls post test. Scale bars = 15 μm (**C, E)**

MCP-1 expression was induced early at P1 (at 4 and 24 h) and only after exposure to LPS or LPS+HI but not HI alone (Figure 7A). At P12, MCP-1 levels were significantly up-regulated, 4 and 24 h after exposure to HI, LPS, or both combined, and this induction was more important than at P1 (4 fold higher at P12, Figure 7A). MCP-1 expression further increased and peaked at 24 h after HI, and at 48 h after LPS+HI exposures. The combined LPS+HI aggression led to more sustained expression than did HI alone (Figure 7A). Using IHC, we confirmed that although MCP-1 levels were induced by LPS and LPS+HI at early time points at P1, no change was detected in intracerebral MCP-1 expression at 48 h and no CD68+ cell was detected (Figure 7B). In contrast, at P12, we detected a significant increase, but only in gray matter, of MCP-1 expression (Figure 7B, C). The distribution of MCP-1 staining at P12 correlated with the lesioned area (described in the Table 1 and shown in Figure 6B) and also with the area displaying CD68+ infiltrating cells (Figure 7D, E). Some CD68+ cells were also detected in the white matter of the hemisphere contralateral to ischemia (data not shown).

Another major regulator of immune cell recruitment is the integrity of the BBB, which can be altered by pro-inflammatory cytokines [41,42]. We found that exposure to HI with or without LPS led to a breakdown of the BBB at P12 only, as shown by albumin extravasation into the brain parenchyma detected 48 h post-HI (Figure 6A). This BBB breakdown was more pronounced within LPS+HI exposed brains than those exposed to HI alone. The areas of BBB breakdown corresponded to the areas of distribution of infiltrating immune cells (Figure 6C, see also 5D and 7D for each cell type distribution separately). The areas of BBB disruption also spatially matched with the infarcted area as seen at 8 days post-HI (Figure 6A-C), and with the location of cytokines/chemokines expression. No staining and no change in albumin extravasation was detected in either gray or white matter - in any of the experimental conditions used - at earlier time points (i.e. 4 h at P12) or at P1. Neuropathological damage induced by LPS+HI at P1 and P12 are presented in the Table 1 and shown in Figure 6B, D. No mast cell infiltration was detected in P1 or P12 brains in any of the experimental conditions used.

## Discussion

Using animal models of brain insults occurring at neurodevelopmental stages equivalent to the *preterm *(P1) and *term *(P12) human brain [37], we showed that exposures to a bacterial endotoxin (LPS) and/or HI led to distinct patterns of neuroinflammatory responses depending on the stage of brain maturation, and on the type of insult. At P1, the neuroinflammatory reaction triggered by HI, LPS or LPS+HI was limited to IL-1β and MCP-1 - with no TNF-α - over-expression, and without any concomitant induction of classic counteracting anti-inflammatory cytokines (see Table 1). IL-1β expression at P1 was more prominent within the cerebral white matter than in the gray matter, thus correlating with predominant white matter damage occurrence previously described under our P1 experimental conditions in rodents [40], and as also typically seen in premature human newborns [4,43]. At P1, anti-inflammatory cytokines' responses were absent (IL-6, IL-10), or even down-regulated (IL-1ra, TGF-β1) under HI, LPS or LPS+HI conditions. Lack of anti-inflammatory response at P1 might deprive the challenged brain of neurotrophic factors - such as TGF-β1, IL-10, IL-1ra and IL-6 - involved in neuronal survival and brain tissue repair following brain injuries [7,44-53]. Therefore, this P1 pro-inflammatory orientation (mainly intracerebral IL-1β and MPC-1 up-regulations) combined with the vulnerability of the immature neural cells might account for the brain damage induced (Table 1). Well described IL-1β neurotoxic effects in conjunction with other noxious mediators released by MCP-1 attracted macrophages, such as matrix metalloproteinases (MMPs) and reactive oxygen species, might be involved in the generation of such LPS+/-HI induced brain damage at P1 [23,54].

At P12, in sharp contrast to P1, both pro- (IL-1β peaking 8-fold higher than TNF-α at 4 h post LPS+HI) and anti-inflammatory cytokines (namely IL-1ra, IL-6, IL-10 and TGF-β1) were over-expressed within brains exposed to HI or LPS+HI (Table 1). Despite the combined induction of pro- and anti-inflammatory cytokines at P12, the balance of pro/anti effects remained strongly oriented towards inflammation, at a neurodevelopmental stage equivalent to the term human brain. HI-, LPS- or LPS+HI-induced chemokine responses (MCP-1 and/or CINC-1), known to derive in part from the activation of the IL-1β pathway [55,56], were much more prominent at P12 than at P1, particularly under LPS+HI conditions compared to HI alone (Table 1). Combined, and likely interacting, IL-1β, TNF-α and chemokine inductions were also associated at P12 (but not at P1) with BBB leakage and massive neutrophil infiltration within HI and LPS+HI damaged brain areas. Known TNF-α myelinotoxicity and IL-1β neurotoxicity, joined to other neurotoxic mediators released by neutrophils, such as MMPs and reactive oxygen species, might well play a central role in the induction of such brain damage (Table 1), as also suggested by neonatal human brain studies [7,18].

After identical endotoxin and/or HI exposures, premature human newborn commonly present patchy areas of white matter damage whereas term newborns display major cortico-subcortical infarcts associated with BBB disruption and leukocyte infiltration [4]. The distinct neuroinflammatory responses to similar aggressions were documented in both preterm- (P1) and term-like (P12) brains, and their mechanistic features might contribute to the strikingly different age-dependent neuropathological differences of perinatal brain damage observed in rodents [38,40,57] as well as between preterm and term human newborns [4].

Systemic adaptive and innate immune responses of the human neonates are known to differ from the adult's one in several ways, such as a bias towards Th2 response, and peripheral blood monocyte stimulation from pathogens leading to higher IL-6, and IL-10 responses, but lower TNF-α expression than in the adults [58,59]. Certain developmental comparisons have already been performed between neonatal and adult neuroinflammatory responses and across neonatal period [60,61]. For instance, a "window of susceptibility" to intracerebral IL-1β exposure was detected in juvenile (2-6 week-old) as opposed to neonatal (P1) or adult (P90) rats, with juvenile rats displaying a peculiar age-dependent hyper-response in term of neutrophil infiltration and neural cell damage [62-64]. Our experimental data further delineate that over a short time frame, the P1 brain reacts differently than the P12 brain following exposure to systemic endotoxin and/or HI aggressions (Table 1). Importantly, the recent demonstration of much lower expression of TLR-4, within P1 rat brain compared to slightly more mature rat brains, might provide a mechanistic explanation to our results [65]. The neuroinflammatory response, even when induced at both developmental stages, was weaker in P1 than in P12 brains in its neurotoxic, as well as neuroprotective components. In agreement with these experimental results, both IL-1ra, IL-1β, and subsequent MMP-9 expressions were shown *in situ*, to be more weakly expressed in preterm than in term damaged white matter from *human *brains [7]. In addition, studies testing the transmigration across barriers of peripheral immune cells showed only modest capacities for circulating leucocytes, such as neutrophils, to migrate into the brain of preterm human newborns as compared to term newborns [59]. This is known to be linked to immature adhesion molecule expression by blood or BBB cells. In addition, the reduced P1 (preterm-like) expressions of MCP-1 and CINC-1 we showed at the most immature stage of postnatal brain development might also contribute to the weak preterm recruitment of systemic cells (neutrophils and CD68+ cells) within the brain in our model, as also observed by others [57,62]. In agreement with the age-dependant level of CINC-1 production we showed, neutrophils have been already shown to be implicated - but only after P7 - in rat ischemic brain damage, with a peak of deleterious effects at P12-P30 [37,62,64]. The fact that we did not detect any immune cell recruitment at P1 as compared to P12 (and P7 as described by others [64,66-68]), shows that the stage of brain and immune system development is crucial in the modulation of neuroinflammatory responses. Recent data disclosed that the neonatal BBB is more mature than previously thought, and that after an inflammatory challenge at P1, the BBB was less permeable than later on [62,69]. This is in keeping with our observations showing that albumin extravasation and leucocytes infiltrations across the BBB were abundant in rat brains exposed to HI, or LPS+HI, at P12, but absent following the same insults at P1. This might also be due to the weaker P1, than P12, neuroinflammatory response avoiding BBB leakage.

## Conclusion

In sum, following HI, LPS and combined LPS+HI, the predominant pro-inflammatory IL-1β response within the brains of preterm- and term-like neonates - whatever the level of anti-inflammatory cytokine response - may have damaging effects at both stages of development. However, the magnitude of the neuroinflammatory response seems to be proportional to the intensity of the IL-1β response as well as to the age-dependent consequences on BBB permeability, chemokines responses, and neutrophil infiltration.

## Competing interests

The authors declare that they have no competing interests.

## Authors' contributions

MEB and SG are co-first authors with equivalent contributions. MEB, SG and KL carried out the experiments and performed statistical analyses. MEB and SG drafted the manuscript. GS conceived the study. GS and SG designed and coordinated the project. GS helped to draft the manuscript. All authors read and approved the final manuscript.
